# Selective Targeting of Virus Replication by Proton Pump Inhibitors

**DOI:** 10.1038/s41598-020-60544-y

**Published:** 2020-03-04

**Authors:** Susan M. Watanabe, Lorna S. Ehrlich, Madeleine Strickland, Xiaofan Li, Veronica Soloveva, Arthur J. Goff, Charles B. Stauft, Sumita Bhaduri-McIntosh, Nico Tjandra, Carol Carter

**Affiliations:** 1 Department of Microbiology and Immunology, Stony Brook University, Stony Brook, NY 11794-5222 USA; 2 Laboratory of Molecular Biophysics, National Heart, Lung, and Blood Institute, National Institutes of Health, Bethesda, MD 20892 USA; 3 Department of Pediatrics, Division of Infectious Diseases and Department of Molecular Genetics and Microbiology, University of Florida, Gainesville, FL 32610 USA; 4 U.S. Army Medical Research Institute of Infectious Diseases, Frederick, MD 21702-5011 USA

**Keywords:** Cell biology, Drug discovery, Microbiology, Structural biology

## Abstract

Two proton pump inhibitors, tenatoprazole and esomeprazole, were previously shown to inhibit HIV-1 egress by blocking the interaction between Tsg101, a member of the ESCRT-I complex, and ubiquitin. Here, we deepen our understanding of prazole budding inhibition by studying a range of viruses in the presence of tenatoprazole. Furthermore, we investigate the relationship between the chemistry of prodrug activation and HIV-1 inhibition for diverse prazoles currently on the market. We report that tenatoprazole is capable of inhibiting the replication of members of the enveloped filo, alpha, and herpes virus families but not the flavivirus group and not the non-enveloped poliovirus. Another key finding is that prazole prodrugs must be activated inside the cell, while their rate of activation *in vitro* correlated to their efficacy in cells. Our study lays the groundwork for future efforts to repurpose prazole-based compounds as antivirals that are both broad-spectrum and selective in nature.

## Introduction

Many enveloped viruses recruit the *e*ndosomal *s*orting *c*omplex *r*equired for *t*ransport (ESCRT) machinery for egress from the infected cell. A P(T/S)AP motif within an encoded structural protein engages the Tsg101 protein in ESCRT-I; this event ultimately delivers ESCRT-III to sites of viral budding where membrane scission releases the viral particle (reviewed in^1,2^). In addition to a P(T/S)AP-binding pocket, Tsg101 also possesses Ubiquitin (Ub)-binding capability that functions in HIV-1 budding, a feature detected through the use of agents identified by random screening of a small molecule library^3^. These agents, tenatoprazole and esomeprazole, are prodrugs used medically as proton pump inhibitors (PPIs) for treatment of acid reflux. Through structural analysis, we showed that they targeted cysteine 73 (C73) in the N-terminal domain of Tsg101, disrupted Ub binding, and prevented Tsg101 localization to the plasma membrane budding site^3^. Tsg101-P(T/S)AP recognition was not disrupted. The inhibition of virus production was suppressed when the pool of endogenous Tsg101 was depleted and replaced with Tsg101 in which Ala was substituted for C73 (C73A) or following addition of N-acetyl Cys as a competitor.

Many enveloped viruses that bud from the plasma membrane, including the retrovirus HIV-1 and the filovirus Ebola (EBOV), exploit the cellular ESCRT machinery to facilitate viral egress, whereupon either Tsg101 or an ESCRT adaptor such as Alix or Nedd4 brings the scission machinery housed in ESCRT-III to the budding site (reviewed in^4,5^). Other enveloped viruses, *e*.*g*., the flaviviruses Dengue (DENV) and Zika (ZIKV), that replicate on the ER membrane, bud into the lumen and exit cells by exocytosis in cellular vesicles. Herpes virus egress includes a combination of transit through the inner nuclear membrane followed by exit via exocytosis. It has been suggested that Alphaviruses [including Sindbis virus (SINV), Semliki Forest virus (SFV) and Mayaro virus (MAYV)] form viral RNA replication/transcription complexes and initiate minus-strand RNA synthesis at the plasma membrane before moving inward to the cytoplasm, possibly via endocytosis, and ultimately bud from the plasma membrane^6^. Interestingly, DENV which like HIV-1 and EBOV requires the PTAP-binding function of Tsg101 for recruitment, does not require the Tsg101-Ub-binding function^7^. In contrast, HIV-1 requires both^3^. Despite suggested use of endocytic machinery, Alphaviruses are reported to be ESCRT-independent^6^. The following observations suggested that Tsg101-Ub binding might regulate Tsg101 recruitment to membrane locations: (*i*) We observed that prazole treatment prevented Tsg101 from accumulating with Gag on the plasma membrane; (*ii*) Prazole treatment differentially inhibited constitutive EGFR recycling to the plasma membrane but not ligand-induced trafficking, which sorts the receptor to an internal degradative compartment^3^, indicating that prazoles disrupt trafficking routes differentially; (*iii*) The nuclear egress complex, conserved among herpesviruses, is comprised of EBV proteins BFRF1/BFLF2. They are co-dependent for their localization to the nuclear rim where they promote budding of immature capsids into the cytoplasm^8^. The Ub E3 ligase Itch, Ub and Alix control BFRF1-mediated modulation of the nuclear envelope through BFRF-1 ubiquitination. Tsg101 co-localizes with BFRF1/BFLF2 at the rim and BFRF1 can be co-immunoprecipitated by Tsg101 antibody. This interaction was not seen in cells expressing BFRF1 alone, indicating that Tsg101 participation may require recruitment by modified BFRF1^9,10^. Tsg101 and Alix are known to be important for MVB biogenesis (reviewed in^4,11^) and MVB formation is considered the topological equivalent of virus budding. Collectively, these observations suggest that just as cell surface receptor ubiquitination signals mobilization of early ESCRT machinery^12^, Tsg101-Ub interaction might signal ESCRT factor delivery to membrane destinations where the early ESCRT interaction would facilitate recruitment of the later-acting ESCRT-III complex.

Here, we demonstrate that, in contrast to the gastric proton-pump target that is accessible to the activated drug at the cell surface, the anti-viral target requires prodrug activation inside the cell. We show that enveloped viruses that exit cells by exocytosis from the ER were resistant to prazole inhibition while those that bud from the plasma membrane were sensitive. Like esomeprazole and tenatoprazole, other currently marketed prazoles also target C73 in Tsg101 and inhibition of HIV-1 budding correlated to the rate of prodrug conversion to the active state. Collectively, these findings support the conclusion that prazole compounds could be effective as broadly acting antiviral agents and suggest that the function they block, Tsg101 Ub binding, may be critical for recruitment of membrane scission apparatus.

## Results

### Antiviral activity requires intracellular prodrug activation

**Figure 1:**
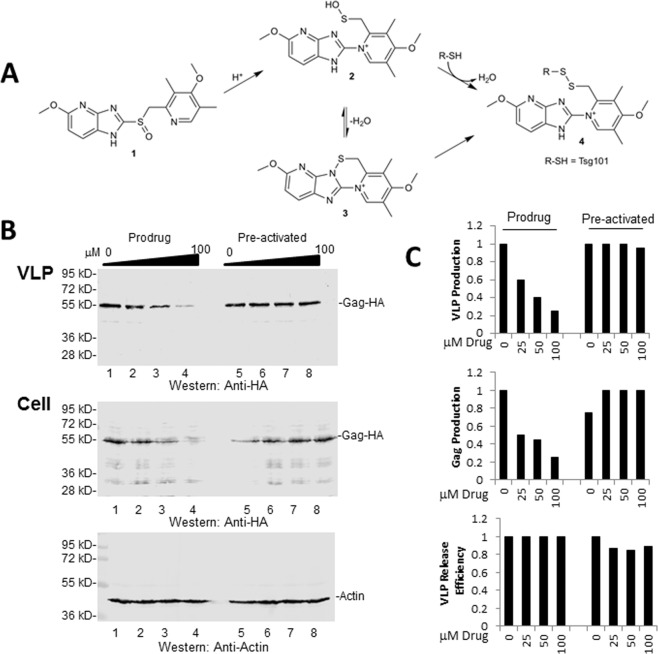
Anti-viral activity requires intracellular prodrug conversion. *Panel A*, Schematic showing conversion of tenatoprazole prodrug (1) to reactive sulfenic acid (2) or sulfenamide (3). Reactivity of 1 is conferred by the centrally-located sulfoxyl group (S=O) which is flanked on the right by a 2-pyridyl group and on the left by an imidazopyridine ring. Molecule 4 shows the position on the activated compound where targets with reactive SH groups (such as C73 on Tsg101, designated as R-SH), bind. Figure is adapted from Strickland *et al*.^3^ *Panel B*, 293 T cells were treated with DMSO (lanes 1, 5) or DMSO plus prodrug (lanes 2–4) or the acid pre-activated compound (lanes 6–8) six hours prior to transfection with DNA encoding HA-tagged HIV-1 Gag. Western blot analysis of isolated VLP (*top*), cell lysate Gag-HA (*middle*), and actin (*bottom*). *Panel C*, quantitative analysis of *Top right*, VLP production normalized to cellular actin; *Middle*, Gag accumulation in the cytoplasm, normalized to actin; *Bottom*, VLP release efficiency, normalized to cellular Gag accumulation plus VLP-associated Gag (*i*.*e*., VLP/(VLP + cellular Gag).

The active form of the prazole that binds covalently to Cys residues in the H^+^/K^+^-ATPase for gastroesophageal reflux disease (GERD) treatment^13,14^ or to C73 in Tsg101 for inhibition of HIV-1^3^ is the sulfenamide derivative of the prodrug. Prazole activation chemistry leading to formation of its sulfenamide derivative is shown in Fig. 1A. The sulfenamide is generated from a multi-step rearrangement of the prodrug^15^. The prodrug prazole is nonpolar and as such is able to cross the lipid bilayer which allows for passage through the plasma membrane and cellular membrane compartments; the positively charged sulfenamide derivative is cell-impermeable^16^. Most likely, the pre-activated form is a mixture of several different compounds. For its role as acid pump inhibitor, the target Cys residues of the prazole sulfenamide derivative is in an ectodomain of the pump^13,14^. Hence, effective *ex-cellulo* activation is actually an attribute of PPI potency against GERD. We speculated that for its role as virus assembly inhibitor, the prazole must first enter the cell, then convert to its sulfenamide derivative and, most importantly, accumulate an effective sulfenamide concentration at the right time in the right place within the cell. Two considerations underlie this speculation: (*i*), HIV-1 Gag synthesis/initial assembly initiates in the cytoplasm^17^ (*reviewed in Maldonado et al*.^18^); (*ii*), Tsg101 distributes between cytoplasm and tubulo-vesicular endosomes^19^. Thus, Tsg101 molecules targeted by sulfenamide are intracellular. To test this notion directly, we compared the effect of treatment with tenatoprazole in prodrug and pre-activated forms on hemagglutinin (HA)-tagged Gag (Gag-HA) virus-like particle (VLP) assembly in 293 T cells. For *ex-cellulo* activation, a volume of a 40 mM stock solution of tenatoprazole was mixed with pH 5.8 sodium phosphate buffer for 17 hr, resulting in formation of a rose-colored precipitate which was subsequently pelleted and re-dissolved in the equivalent volume of DMSO. The 40 mM prodrug and pre-activated tenatoprazole stock solutions were used in parallel inhibition assays. Cell extracts and VLPs were prepared as described in Materials & Methods and samples were examined by Western analysis. The Western blot results are shown in Fig. 1B. In Fig. 1C, the amount of Gag detected in isolated VLPs or in the cytoplasm and the release efficiency is shown. Addition to the media of tenatoprazole in prodrug form (lanes 2–4) resulted in dose-dependent inhibition of VLP production compared to the DMSO control (lane 1). In contrast, samples treated with the pre-activated mixture (lanes 6–8) produced amounts of VLPs comparable to the DMSO control (lane 5). This finding indicated that prodrug entry into cells is requisite for anti-viral efficacy. The cell lysate also showed a drug concentration-dependent decline in Gag accumulation in samples treated with the prodrug (lanes 2–4) but not in the samples treated with the pre-activated mixture (lanes 6–8). The diminished intracellular Gag accumulation in cells exposed to the prodrug was reported previously and found to reflect Gag mis-sorting to degradative compartments^3^. Quantitation of VLP release efficiency indicated no change, suggesting that the mis-sorted Gag population derived from the pool that normally produced released VLPs. Thus, prodrug activation inside cells diverts Gag from the productive trafficking pathway that leads to plasma membrane assembly and release of viral particles.

### Tenatoprazole susceptibility of viruses unrelated to HIV-1

As tenatoprazole blocks Tsg101 Ub binding rather than Tsg101 interaction with PTAP-containing sequences, we reasoned that susceptibility to the drug might not be limited to viruses that employ the motif to recruit the protein and therefore examined unrelated viruses for prazole sensitivity. We focused in particular on viruses that cause widespread morbidity, are emerging or re-appearing, and for which few or no drugs currently exist. This list includes the Mayaro (MAYV), Dengue (DENV) and Zika (ZIKV) viruses, which are emerging or are recently appearing mosquito-borne pathogens; Ebola virus (EBOV), the re-appearing causative agent of a severe, often fatal hemorrhagic illness, and Epstein Barr virus (EBV), the etiological agent of a common latent infection whose reactivation following organ transplant is a significant cause of morbidity and mortality. Poliovirus (PV) was tested as a control. It replicates on intracellular host membranes but its capsid possesses no envelope. Each virus was assayed using cell lines and conditions considered standard for the entity (typically 293 T or HeLa cells). Tenatoprazole cytotoxicity in these cells (CC_50_) was estimated to be 129 μM [95% confidence interval (CI) = 156–205 μM] for HeLa and 123 μM (95% CI = 99.8–139.4 μM) for 293 T, based on the WST-1 assay for metabolic function (Supplemental Fig. 1).

Tsg101 and/or ESCRT has been reported to play a role in replication of DENV^7^, EBOV^20,21^, and EBV^9,22^. Of most significance to the current investigation, the exocytic egress of flaviviruses like DENV, Japanese Encephalitis Virus (JEV) and by extension ZIKV, which bud into the lumen of the ER and exit cells in vesicles formed in the secretory pathway, does not require the Tsg101 Ub-binding function^7^. As we recently demonstrated that this function is required for HIV-1 budding as revealed by the proton pump inhibitors that concomitantly disrupted it and arrested budding, we hypothesized that the Tsg101 Ub-binding function was superfluous for ER budding but necessary for egress at the plasma membrane location. This hypothesis predicted that DENV and ZIKV, which assemble on the ER, should be resistant to the prazole while EBOV, which buds from the PM, should be susceptible.

**Figure 2:**
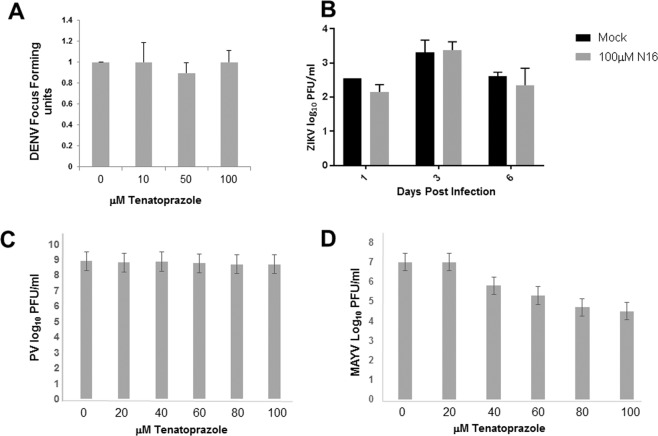
Flavi- and Picornaviruses exhibited tenatoprazole resistance; MAYV exhibited susceptibility. 293 T cells were pre-treated for 6 hr (DENV, ZIKV, MAYV) or 7 hr (PV) with media containing either DMSO (0) or the indicated concentration of tenatoprazole. Cells were then infected with *Panel A*, the synthetic DENV 2 strain 16681; *panel B*, ZIKV; *Panel C*, the Mahoney strain of PV at MOI = 0.01; *Panel D*, MAYV at MOI ~0.001. After two hours, the tissue culture media was removed and replaced with fresh treatment media. After two days, the media was collected and the virus titer was measured by focus forming assay where foci were developed with 4g2 primary anti-flavivirus antibody (DENV: the y axis shows the relative change compared to the DMSO control) or plaque assay in Vero cells (ZIKV, PV, MAYV). Plaques were visualized by staining with crystal violet. The y-axis show the virus titers in plaque-forming units. Error bars equal 1 SD.

To investigate, DENV and ZIKV production in the presence and absence of the drug was tested on 293 T cells, with infectious virus output measured by focus forming (DENV) or plaque forming assays (ZIKV) in Vero cells. As shown in Fig. 2A, DENV replication exhibited no tenatoprazole susceptibility at concentrations as high as 100 μM (panel A). Similarly, ZIKV exhibited <2-fold reduction in infectious virus output following exposure to 100 μM, as measured by plaque assay (Fig. 2B). This reduction was not maintained at three or six day sampling and is not considered significant. As predicted, PV exhibited no tenatoprazole susceptibility, as revealed by plaque assay, at concentrations as high as 100 μM (Fig. 2C). Interestingly, tenatoprazole treatment reduced MAYV titer in a dose-dependent manner up to >2 log_10_ at 100 μM (Fig. 2D).

**Figure 3:**
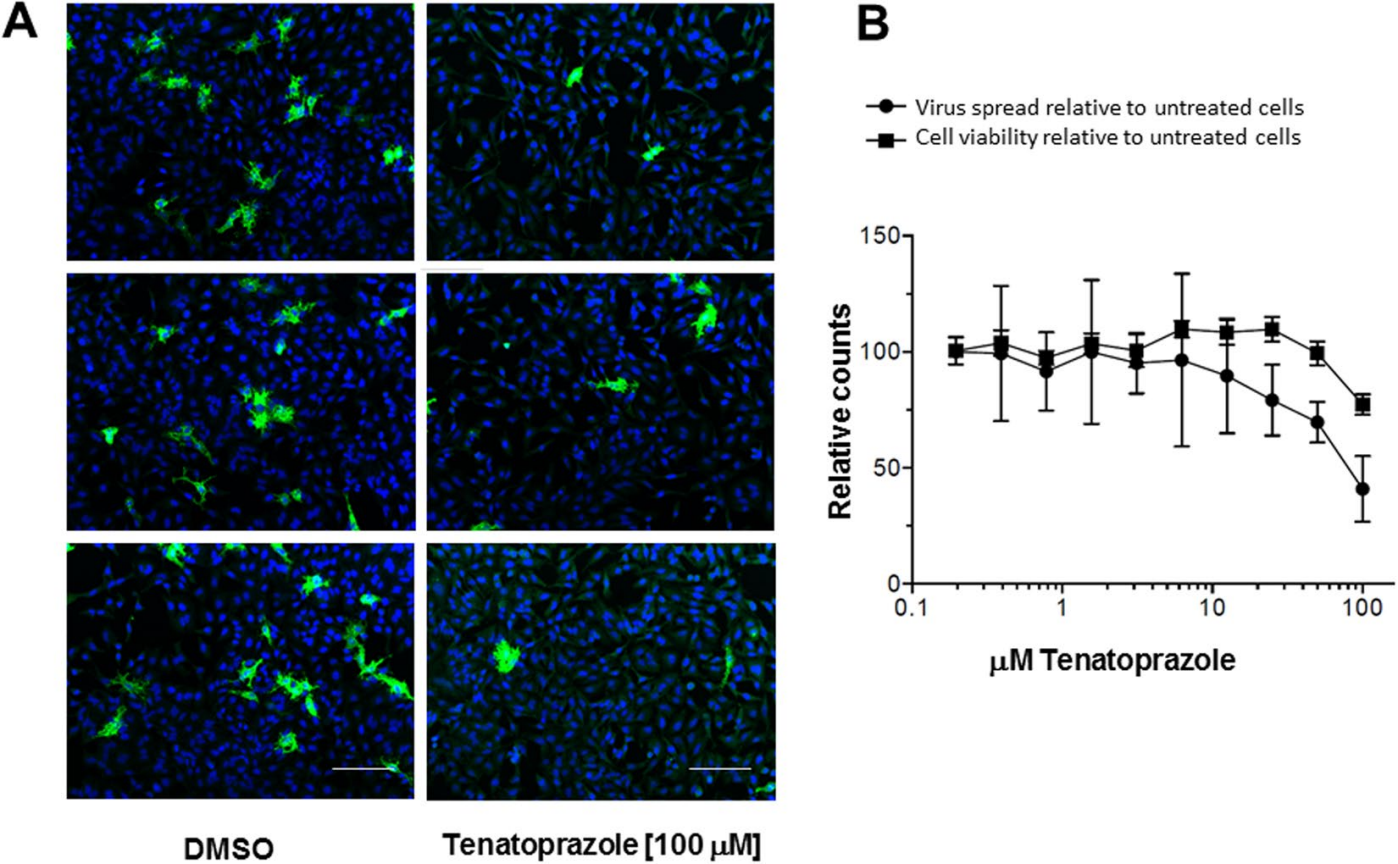
Susceptibility of EBOV replication to tenatoprazole. HeLa cells were pre-treated for two hours with media containing either DMSO (0) or ten concentrations of tenatoprazole up to 100 μM in DMSO. EBOV (Zaire) was added at MOI = 0.5. After 24 hr, infection was stopped by fixing the cells and virus spread was determined by immuno-staining for viral glycoprotein (GP). *Panel A*, Triplicate samples showing GP-staining signal after 24 hr. *Left*, cells treated with DMSO alone; *Right*, cells treated with 100 μM tenatoprazole in DMSO. Scale bars equal 50 microns. *Panel B*, Inhibition of virus infection quantified based on reduction in virus spread determined at each concentration (N = 8 for each concentration). Cell viability was determined from the number of nuclei per well in treated samples compared to infected but untreated controls. Virus spread was calculated relative to cells with no drug. Error bars equal 1 SD.

The EBOV VP40 protein which, all by itself directs assembly and budding from the plasma membrane, contains overlapping PTAP and PY Late domains^21,23^ and an LYP(X)_n_L sequence^24^ (*i*.*e*., motifs required for recruitment of Tsg101, Nedd4 and ALIX proteins, *respectively*). Again supporting the hypothesis, the results indicate that the drug reduced the spread of EBOV in cultures of HeLa cells, as monitored by immunostaining for the viral glycoprotein (Fig. 3A). Inhibition exhibited an EC_50_ = 80 μM, a concentration at which >80% of the cells appeared viable (Fig. 3B).

**Figure 4:**
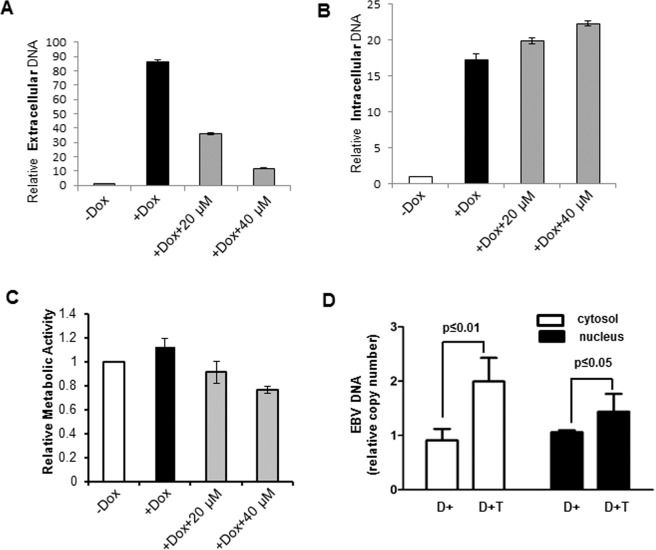
EBV replication is tenatoprazole-sensitive. Latent EBV was reactivated in CLIX-FZ cells by addition of doxycycline in the presence of DMSO (*black bars*) or DMSO plus tenatoprazole (20 µM or 40 µM, *grey bars*); non-treated cells are shown as white bars. After 72 hr, cells and conditioned media were harvested. *Panel A*, extracellular EBV DNA measured in pellet fraction of conditioned media. *Panel B*, intracellular EBV DNA measured in lysed cells. EBV DNA was determined by quantitative-PCR (qPCR) for the EBV *BALF5* gene. EBV DNA copy number from each preparation was normalized to the DMSO-treated control. *Panel C*, metabolic activity of cells in the presence of tenatoprazole. The cells were grown for 24 hr in media containing the indicated concentrations of doxycycline and tenatoprazole followed by assessment of metabolic activity assessed using the WST-1 assay. *Panel D*, fractionation of intracellular DNA. CLIX-FZ cells were treated with doxycycline plus DMSO (D+) or doxycycline plus tenatoprazole (D+T), harvested 72 hr later, and separated into nuclear (*black bars*) and cytosolic (*white bars*) fractions. DNA was extracted from each fraction and the relative number of EBV genomes from each fraction was determined by qPCR using primers directed towards the EBV *BALF5* gene. The number of genomes recovered was significantly different as judged by the Students t-test, two-tailed. Error bars equal 1 SD.

In contrast to HIV-1, DENV, ZIKV, MAYV and EBOV, which launch lytic infections, EBV replication takes place mainly in the nucleus and is mostly latent. Following activation of the lytic phase, an immature form of the virus buds through the inner nuclear membrane (INM) to complete the maturation process in the cytoplasm. Mature virus leaves the cell through an exocytic pathway, however previous studies showed that ubiquitination events faciliate the initial step, *i*.*e*., budding through the inner nuclear membrane^10^. To test for prazole sensitivity, EBV lytic activation was triggered by exogenous expression of ZEBRA, the EBV latency-to-lytic switch protein, using doxycycline in CLIX-FZ, an engineered Burkitt lymphoma cell line^25^. Tenatoprazole impact on EBV production was assayed using quantitative PCR (qPCR). Simultaneous treatment with tenatoprazole at concentrations of 20 µM and 40 μM resulted in a dose-dependent reduction in the number of released (*i*.*e*., extracellular) EBV particles compared to the yield from cells treated with vehicle plus doxycycline (Fig. 4A). Commensurately, the intracellular EBV load increased (Fig. 4B), indicating that reactivation from latency had proceeded unperturbed but the assembled capsids were trapped inside the drug-treated cells. The host CLIX-FZ cells exhibited some impairment of metabolic capacity in the 40 μM concentration range (Fig. 4C). However, as this diminished cell capacity did not reduce the total intracellular EBV load, it seems unlikely that the observed inhibition of virus release was attributable (solely) to cytotoxicity. To assess the site of entrapment, nuclear and cytosolic fractions were prepared from control and drug-treated reactivated CLIX-FZ cells and re-assayed for EBV DNA by qPCR using primers directed towards the EBV *BALF5* gene that encodes the viral polymerase. As shown in Fig. 4D, EBV DNA was dose-dependently trapped in both compartments. The results indicate that tenatoprazole did not inhibit virus replication but rather its egress. Collectively, the results (Table 1) indicated that tenatoprazole susceptibility was broad yet selective.

**Table 1:** Tenatoprazole Susceptibility of Viruses Unrelated to HIV.

Virus	Cell line	Budding Site	Virus Production	Component Assayed	Assay
DENV	293 T	Endoplasmic reticulum	Resistant	Infectivity	FFU
EBOV	HeLa	Plasma membrane	Susceptible (1.6)	GP protein	IS
EBV	CLIX-FZ	Nuclear membrane	Susceptible (~4)	*BALF5*	qPCR
HIV-1	293 T	Plasma membrane	Susceptible (2)	Gag p24	WB
MAYV	293 T	Plasma membrane	Susceptible (2.5)	Infectivity	PFU
PV	293 T	None	Resistant	Infectivity	PFU
ZIKV	293 T	Endoplasmic reticulum	Resistant	Infectivity	PFU

Tenatoprazole susceptibility was assessed for each virus using an assay standard for the field. Abbreviations are: focus forming units (FFU), immunostain (IS), quantitative PCR (qPCR), Western blot (WB), and plaque forming units (PFU). Virus production was determined as resistant or susceptible, with selectivity Index indicated in parentheses. Selectivity index is defined as the ratio of cytotoxic concentration to effective concentration (CC_50_/EC_50_). CC_50_ is the compound’s concentration (µM) required for the reduction of cell viability by 50%; EC_50_ is the compound’s concentration (µM) required for the reduction in the assay of virus production by 50%.

### Related prazoles differ in their conversion rates to their active form(s)

**Figure 5:**
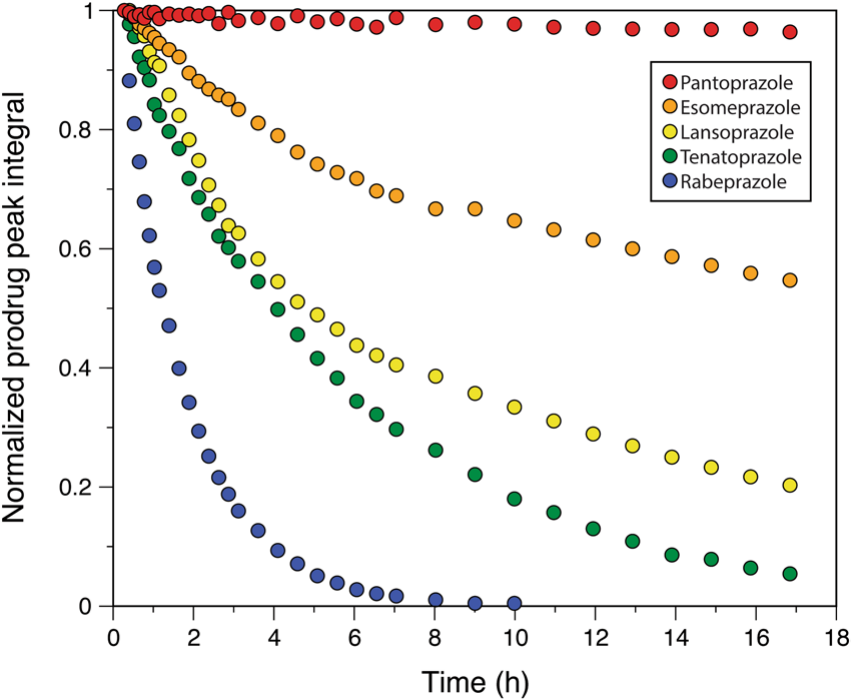
Prazole prodrugs are converted to sulfenamide derivatives over time in acidic environment *in vitro*. Prodrug remaining in pH 5.8 buffer solution at time intervals over a 17 hr examination period was detected using 1D proton NMR spectra analyses. Peak heights were normalized to the initial reading for each prodrug and plotted against time.

As a biochemical group prazoles^26^ share as core structures benzimidazole and pyridine and can be divided into two groups based on their basic structure as imidazopyridine (tenatoprazole designated as N16 in Strickland *et al*.^3^) and benzimidazoles (rabeprazole lansoprazole, esomeprazole, and pantoprazole). All compounds share the same acid-mediated activation chemistry in cells (Fig. 1A). As their structures differ and, as we determined that anti-viral activity required prodrug conversion to the active form(s) inside the cell (Fig. 1), it was important to determine how these properties influenced their anti-viral efficacy. Previously, we showed that tenatoprazole bound covalently to residue C73 of Tsg101 using a disulfide bond^3^. We also solved the structure of the tenatoprazole-Tsg101 complex using NMR spectroscopy, which detailed the binding interaction between the two compounds, including the covalent disulfide bond. Esomeprazole, pantoprazole, rabeprazole, and lansoprazole have similar chemical structures to tenatoprazole and all induce the largest chemical shift perturbations around residue C73 of Tsg101, indicating that they also bind covalently to this residue. We therefore determined the rate at which the prodrug forms of tenatoprazole, esomeprazole, lansoprazole, rabeprazole and pantoprazole were converted to their respective sulfenamide derivatives *in vitro*. Using LC-MS, we determined that a compound similar to the sulfenamide derivative (Fig. 1A) was formed upon addition of the prodrug to mildly acidic aqueous buffer (Supplemental Fig. 2), as previously shown for rabeprazole^27^. Using NMR spectroscopy, we could follow the time-dependent disappearance of prodrug proton peaks as a surrogate for activation under similar conditions as the LC-MS experiment. As shown in Fig. 5, rabeprazole had the highest rate of conversion, completely disappearing in less than ten hours. Pantoprazole remained predominantly as a prodrug (96%) by the end of the 17 hour time course. Esomeprazole and tenatoprazole, which differ by only one atom, had quite different rates with ~55% and ~5% prodrug, respectively, remaining at 17 hours. Lansoprazole was intermediate at 20%. The observed conversion rates (rabeprazole > tenatoprazole > lansoprazole > esomeprazole > pantoprazole) are in agreement with that obtained at pH 5.1 by Shin *et al*.^15^. In all cases, we observed that the concentrations of prodrug and sulfenic acid were inversely proportional to each other, validating our following of the prodrug peak in the NMR experiments.

Chirality at the sulfoxide center is a characteristic of all the prazoles examined in this study. Chirality as a factor in therapeutic efficacy reflects the stereo-selectivity of metabolic clearance of prazoles^28^ but whether chirality influences conversion rate is not clear. We therefore examined this property, as the sulfoxide group can have both *S*- and *R*-enantiomers and unless specific measures are taken to produce or isolate one enantiomer, most available prazoles are racemic mixtures. Studied in their racemic mix were: tenatoprazole, lansoprazole, rabeprazole, and pantoprazole. Esomeprazole (omeprazole *S*-enantiomer) and lansoprazole (lansoprazole *R*-enantiomer) were tested as enantiomers. We detected no significant difference in rates for dexlansoprazole and lansoprazole (Supplemental Fig. 3A).

**Figure 6:**
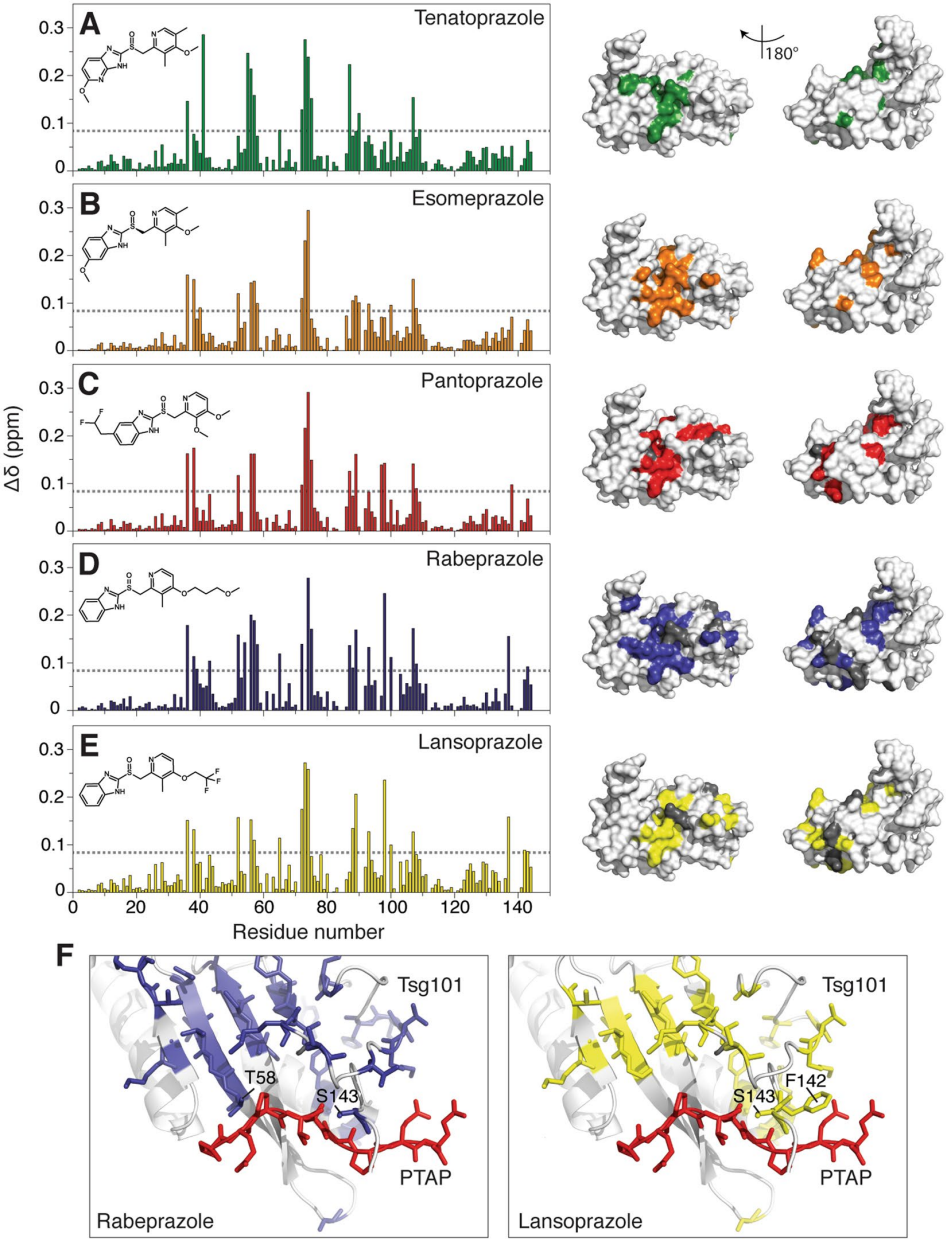
Identification of prazole target in Tsg101 UEV domain. Chemical shift perturbations in Tsg101 UEV complexed with prazole derivatives are shown in *Panels A to E*. Prazoles were mixed with purified Tsg101 UEV domain protein and chemical shift perturbations measured after covalent attachment and subsequent buffer exchange. *Left inset*, prazole structure; *Right*, chemical shift perturbations. Perturbed residues are marked in color on the front and backside of the UEV domain structure: *green*, tenatoprazole; *orange*, esomeprazole; *red*, pantoprazole; *blue*, rabeprazole; *yellow*, lansoprazole. Residues that cannot be assigned or that disappear in the bound spectrum are marked in grey. The cut-off for large chemical shift perturbations was standardized as 0.084 ppm (1.5 standard deviations from zero for tenatoprazole chemical shift perturbations). *Panel F*, Models showing residues in PTAP-binding pocket perturbed by prazole binding (PDB ID 1M4Q, Pornillos, *et al*.^26^).

It was previously shown that Tsg101 binds to tenatoprazole through a covalent bond at residue C73, with the orientation of the ligand determined by interactions with neighboring residues (D40, S41, T56, W75, K90), as determined by intermolecular carbon isotope-filtered nuclear Overhauser effect (NOE) NMR spectra and chemical shift perturbations^3^. Large chemical shift perturbations or peak broadening indicate a change in chemical environment associated with binding or binding-induced structural change and was used here to detect differences in binding between the five prazole compounds and Tsg101 **(**Fig. 6). Overall, it was found that the three new compounds bind to Tsg101 in a similar manner as tenatoprazole, forming a covalent disulfide linkage with C73 and contacting many of the same residues. Table 2 shows residues that undergo large chemical shift perturbations or peak broadening upon addition of the five prazole compounds. Affected residues that are common to all drugs are indicated in bold font. There are some subtle differences to note: Tenatoprazole is unique in that it contains nitrogen in place of the carbon in the benzimidazole ring in the other prazoles. Interestingly, tenatoprazole is the only compound to form a hydrogen bond with the backbone amide of S41 (as shown by the characteristically large chemical shift change of 0.29 ppm versus an average of 0.03 ± 0.02 ppm for the other ligands). Instead, the other prazoles induce large chemical shift perturbations in residues V38 and L52 (not observed for tenatoprazole), indicating that the conversion from an imidazopyridine ring to a benzimidazole encourages the ligand to interact with more hydrophobic residues in favor of a hydrogen bond to S41, and may therefore have a slightly different orientation. Secondly, rabeprazole and lansoprazole have bulky additions to their pyridine ring and induce further chemical shift perturbations in residues K98 and H102, and around the C-terminus (S135, E137, S143). Figure 6F, shows the proximity to the PTAP-binding pocket of residues perturbed due to the additional bulk of rabeprazole and lansoprazole. The changes in this region are likely the result of structural changes in the UEV domain rather than the direct impact of ligand binding, since they are far from the tenatoprazole binding site. As noted above, no significant difference in conversion rate was detected for complexes with lansoprazole and its enantiomer dexlansoprazole and these showed essentially identical chemical shifts, which is consistent with formation of identical covalently-bound derivatives upon activation of the two prodrugs (Supplemental Fig. 3B). These findings support the conclusion that, as a biochemical class, prazoles target the Tsg101 Ub-binding pocket region, specifically, C73.

**Table 2:** Chemical Shift Perturbations and Broadened Peaks Observed Upon Prazole Binding to Tsg101.

Tenatoprazole	Esomeprazole	Pantoprazole	Rabeprazole	Lansoprazole
**K36**	**K36**	**K36**	**K36**	**K36**
	V38	V38	V38	V38
			L39	
	D40			
S41				
			V43	
	L52	L52	L52	L52
		N54^a^	N54	
**L55**	**L55**^a^	**L55**^a^	**L55**^a^	**L55**^a^
**T56**	**T56**	**T56**	**T56**	**T56**
**G57**	**G57**	**G57**	**G57**	**G57**
	T58		T58	
G65			G65	G65
**I72**	**I72**	**I72**	**I72**	**I72**
**C73**	**C73**	**C73**	**C73**^a^	**C73**
**L74**	**L74**	**L74**	**L74**	**L74**
W75		W75	W75	
C87		C87	C87	
	F88		F88	F88
	V89	V89	V89	V89
K90	K90		K90^a^	K90^a^
	S93		S93	S93
		T96^a^	T96^a^	
		I97		
		K98	K98	K98
			T99^a^	T99^a^
G100	G100		G100	G100
			K101^a^	
			H102^a^	H102^a^
**G107**	**G107**	**G107**	**G107**	**G107**
	K108	K108	K108	
I109				
			F135^a^	F135^a^
			D137	D137
		E138		E138^a^
				V141^a^
				F142
			S143	S143

Prazole binding was determined by NMR. Residues that were broadened beyond detection upon addition of ligand are indicated by superscript (^a^). Bold font indicates affected residues common to all drugs tested.

### Prazole conversion rates predict inhibition of HIV budding

**Figure 7:**
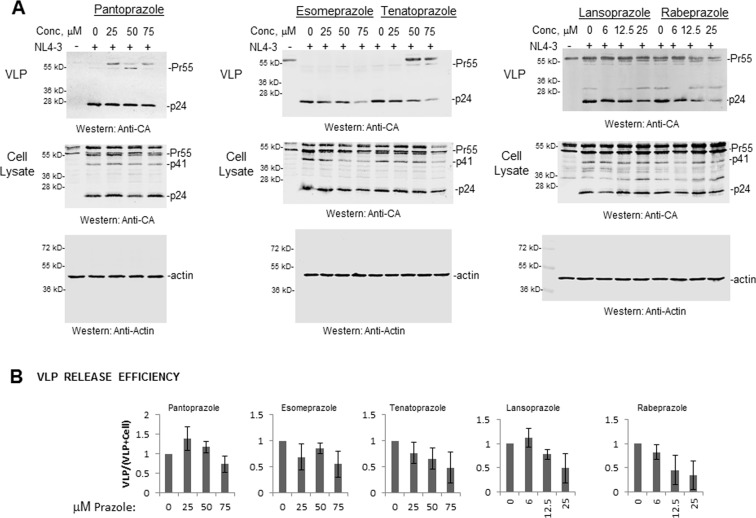
Prazole potency against HIV-1. 293 T cells were co-transfected with pNL4-3Δ Env and pIII Env plasmids about six hours after addition of prazole compound in the 0–75 μM concentration range. VLPs and cell lysates were prepared 24 hr after transfection. *Panel A*, Western analysis of viral particles and cell lysates for CA p24-related proteins. *Panel B*, Quantitation of virus release efficiency based on CA p24 signal. Error bars equal 1 SD.

The five prazoles, whose rates of conversion and binding to the Tsg101 UEV domain were characterized in Figs. 5 and 6, were compared for potency in inhibiting HIV-1 production. All were tested in the 0–75 μM range. Cytotoxicity (CC_50_) for these prazoles is shown in Supplemental Fig. 4. The effect of these prazoles on virus production is shown in Fig. 7. Pantoprazole (lanes 2–4) exhibited negligible inhibitory activity. As previously reported, esomeprazole (*panel* A, lanes 7–9) was less efficacious than tenatoprazole (lanes 12–14) while lansoprazole (lanes 16–18) exhibited greater potency than tenatoprazole. Rabeprazole provided the strongest inhibitory effect (lanes 20–23). Potency ranking was therefore as follows: pantoprazole (EC_50_ > 75 μM), esomeprazole (EC_50_~75 μM), tenatoprazole (EC_50_~50 μM), lansoprazole (EC_50_~25 μM) and rabeprazole (EC_50_~15 μM) (Table 3). Comparison with the results shown in Figs. 5 and 7 indicated that antiviral activity correlated directly with the prazole conversion rate and possibly also with bulky side group addition.

**Table 3:** HIV-1 Prazole Susceptibility.

Prazole	Cytotoxicity (CC_50_)	Approximate Effective Concentration (EC_50_)	Selectivity Index
Esomeprazole	75 μM	75 μM	1.0
Lansoprazole	50 μM	25 μM	2.0
Pantoprazole	40 μM	>75 μM	<0.5
Rabeprazole	150 μM	15 μM	10
Tenatoprazole	125 μM	50 μM	2.5

HIV-1 prazole susceptibility was assessed by comparing CC_50_ (concentration at which cells exhibited 50% metabolic activity of control as assayed with WST reagent (see Materials & Methods) and the approximate EC50 (concentration at which VLP production was reduced to 50% of control as assayed by Western analysis). The selectivity index (CC_50_/EC_50_) ranks relative theoretical effectiveness as an HIV-1 antiviral agent.

We conclude that a direct relationship exists between formation of the sulfenamide compound that attacks C73 in the Tsg101 UEV domain and prazole inhibitory potential.

## Discussion

Here, we report that prazoles, approved for use as PPIs, also exhibit potential for antiviral therapy. For this application, the prodrug required conversion inside cells. HIV-1, MAYV, EBOV and EBV replication were tenatoprazole sensitive while DENV, ZIKV and PV replication were resistant. Further testing employing HIV-1 as a model revealed its susceptibility to additional prazoles with inhibition generally correlating to the rate of pro-drug conversion to the active sulfenamide derivative. In all cases, the prazoles targeted the Tsg101 UEV domain C73 residue, indicating that they inhibited Tsg101 function through the same mechanism. It should also be noted that previous studies demonstrated that esomeprazole and omeprazole inhibited entry of a lentivirus-based pseudotyped particle using the EBOV Env (G) protein^29^. As noted already, both the chiral esomeprazole and the racemic omeprazole would be converted to the same achiral active sulphenamide derivatives which are expected to target Tsg101 identically. In the case of the pseudotyped virus particle, inhibition was observed and attributed to the off-target inhibitory activity on endosomal vacuolar ATPase^29^. In our studies, esomeprazole had no significant inhibitory effect at concentrations up to 100 μM but tenatoprazole showed some efficacy (EC_50_ = ~80μM). These results are consistent with those previously reported for the pseudotyped virus tested in 293T cells (esomeprazole EC_50_ = 50 μM)^29^.

Tabata *et al*. proposed that DENV recruits Tsg101, an early acting ESCRT, to its budding site on the ER membrane to function as an adaptor to recruit downstream ESCRTs, the CHMP2/3 and CHMP4 proteins needed for budding^7^. They suggested these factors comprised a “common core machinery” distinct from that involved in cytokinesis or MVB vesicle formation. We hypothesize that viruses like HIV-1 that bud from the plasma membrane recruit Tsg101 to access downstream membrane scission machinery comprised of CHMP2, 4 and other ESCRT-III factors. As part of this, they may “tailor” the core machinery to exploit features that facilitate Tsg101 recruitment, Vps4-mediated membrane scission and/or ESCRT factor recycling, and MVB formation/function. In tailoring the core machinery, different viruses would employ subsets of the ESCRT factors, ESCRT factor isoforms and/or different adaptor proteins to facilitate their trafficking to the egress destination. Possibly, they mimic the plasticity inherent within the machinery. For example, although all Chmp4 isoforms inhibit both HIV-1 release and cytokinesis to some degree, Chmp4b exhibits the most potent inhibition of viral budding while Chmp4c was found to be a much more potent inhibitor of cytokinesis than either Chmp4a or Chmp4b^30^. This differential inhibition of HIV-1 budding and cytokinesis by heterologous Chmp4 suggests that the different functions have “tailored” their utilization of the different ESCRT factors. Indeed, although there is only one form of Tsg101, theoretically, it could exist as part of several unique ESCRT-I complexes, each tailored to optimize a particular role. Human ESCRT-I is a tetramer which, in addition to Tsg101, contains one of two isoforms of VPS28 (generated by alternative splicing), one of four Vps37 isoforms, and one of three Mvb12-related proteins. Depletion of one of the Mvb-related forms prevents MVB sorting of EGFR but not cytokinesis or viral budding^31,32^. Thus, the ESCRT-I subset that normally includes this form has been tailored for a particular cellular function. Depending on their needs, some viruses might similarly tailor an ESCRT-I complex to ultimately provide the best mimicry of the MVB's ability to stimulate membrane curvature away from the cytoplasm, creating the topological equivalent of the membrane evagination events that form virus buds. Viruses such as HIV-1 and EIAV have been shown to utilize a limited subset of the ESCRT-III factors^33,34^. Perhaps, by synthesizing all of their proteins on the ER, remodeling the ER to incorporate both viral and host factors, assembling viral particles on the remodeled ER membrane and then exiting via the secretory pathway, Flaviviruses have optimized the components necessary for membrane remodeling at this site. Tabata *et al*. were unable to identify a need for Vps4 or alternate AAA ATPases. Interestingly, they showed that the PTAP binding ability was important and reported that NS3 bound directly to Tsg101 but no motif has been identified in the NS3 protein. We hypothesize that the requirement for the PTAP-binding but not the Ub-binding function of Tsg101 indicates that Tsg101 recruitment can be a multi-step process for some viruses, such as HIV-1, but not others.

What might determine a virus’ susceptibility to inhibition by prazoles, or the need for more than “common core machinery”? In Supplemental Fig. 5, we show that an HIV-1 Gag mutant, bearing a disrupted PTAP motif (P7L-Gag) and whose egress is therefore independent of direct Tsg101 binding, nevertheless exhibited prazole sensitivity (panel A). It is well-established that the stimulation of release observed following ALIX overexpression^30,35–37^ is independent of Tsg101^38^. Supporting the specificity of prazole targeting to Tsg101, Alix rescue of P7L-Gag was tenatoprazole resistant, averaging 2–3 fold in the concentration range to which the WT is susceptible (panel B). The Alix-mediated rescue includes stimulation of p25 to p24 maturation and this event was also resistant to tenatoprazole inhibition, averaging ~3-fold in the presence or absence of the drug (panel C). It was previously established that Nedd4–2 (also called Nedd4L) and some other Nedd4 isoforms can potently rescue the release of HIV-1 Gag with a disrupted PTAP motif^37,38^. Previous studies showed that Gag was the target of these Nedd4 enzymes^37^. Nedd4 operates upstream of Tsg101 since Tsg101 depletion abolished the ability of the enzyme to rescue the HIV-1 PTAP mutant^38^. We interpret this to mean that, although Gag-Tsg101 binding is not occurring, Tsg101 is still needed in the cell for the budding process. We hypothesize that prazole susceptibility reflects a requirement for Nedd4-mediated modification of Tsg101, thereby permitting Gag to recruit Tsg101 from its inactive form in the cytosol to the plasma membrane. Elsewhere (Strickland, *et al*., *manuscript submitted*), we show that the UEV domain in Tsg101 binds Lys63-linked di-Ub (K63-Ub_2_), a Ub-derivative formed by Nedd4^39^. We also show that Gag^PTAP^-mediated recognition of Tsg101 requires the determinants in the UEV domain that bind K63-Ub_2_. Rabeprazole can disrupt the interaction. Thus, we hypothesize that Tsg101, like other ESCRT factors, exists in active and auto-inhibited states and that the susceptibility to prazole inhibition of any virus is based on a need to activate the factor and mobilize ESCRT-I.

Collectively, the results described here: (*i*) show that the antiviral activity applies broadly to several families of human pathogens and yet may selectively target a property of the ESCRT machinery that is common only to budding at certain cellular membranes; (*ii*) extend the demonstration of antiviral activity to additional members of the prazole class and provide evidence that all prazoles tested target the Ub-binding pocket at the N-terminus of Tsg101 and thereby operate through the same molecular mechanism.

Cells of hematopoetic lineage, notably lymphocytes, are important in the pathogenesis of viruses. Prazoles access these cells when circulating in plasma. Commonly marketed PPIs taken at a single daily dose achieve plasma concentrations ranging from 0.1–23 μM^14^ which may be increased though use of a single enantiomer or by extended release delivery. Our study demonstrates that, in the context of virus production in tissue culture, a tenatoprazole concentration in this order of magnitude was inhibitory in two cases (HIV-1 and EBV). Thus, PPIs as currently administered, were efficacious as antivirals at concentrations feasible to achieve in plasma. Although we know of no focused or systematic study, it is highly likely that PPI use has been unintentionally employed supportively in several clinical settings. As our study mainly examined just one prazole (tenatoprazole) against a panel of viruses and employed assays that assessed different stages in the viral life cycle and in different tissue culture cell environments, the appraisal of susceptibility here is not intended to be definitive. The inhibitory potential against any of the viruses might be improved by selection of a different prazole compound. Drug repositioning or repurposing is a growing trend as a discovery strategy which is both cost-effective and permits leveraging of the proven safety profile. In the case of PPIs, there are already several studies exploring the compounds for cancer^40^ and genetic disease^41^ therapeutics. Our findings provide proof-of-concept that prazoles are promising candidates for repurposing as antiviral agents.

## Methods

### Compounds

Compounds were purchased from Sigma, Toronto Research Chemicals and Selleck Chemicals. Stock solutions were prepared in DMSO (100%) and stored in aliquots at −80 C.

### Prazole degradation rate

Stock solutions (20 mM) of each prazole prodrug were prepared in deuterated DMSO (DMSO-d_6_) and diluted in NMR buffer (20 mM potassium phosphate, 50 mM NaCl, pH 5.8), to a final prazole concentration of 2 mM. Proton spectra of prodrugs were acquired at intervals during a 17-hour experiment. Esomeprazole magnesium hydrate, rabeprazole sodium, pantoprazole sodium hydrate, lansoprazole (Sigma-Aldrich), dexlansoprazole, and tenatoprazole (Toronto Research Chemicals) were each dissolved in deuterated DMSO (DMSO-d_6_) to make 20 mM stock solutions. 50 µL of stock solution was added to 450 µL of NMR buffer (20 mM potassium phosphate, 50 mM sodium chloride, pH 5.8) to a final prazole concentration of 2 mM. Samples were mixed inside the NMR tube (Wilmad 535-PP) using a 100 µL calibrated pipet (Drummond Scientific Company) attached to a 200 µL pipette via rubber tubing. All NMR experiments were started exactly 13 minutes after sample mixing, which allowed for locking (to DMSO-d_6_), tuning, and shimming. Proton spectra were acquired with 1.2 second water pre-saturation and increasing numbers of scans over time (8 × 256 scans, 8 × 512 scans, 8 × 1024 scans, 10 × 2048 scans, which corresponded to experiment times of 7.5 min, 15 min, 30 min, and 60 min, respectively, and a total experiment time of 17 hr). Plotted time points are the mid-point of each experiment, taking into account initial experiment setup and sample mixing. To follow degradation, resonances were chosen that were far from the water signal and non-exchangeable, (esomeprazole, 1.844 ppm (methyl); rabeprazole, 8.009/7.999 ppm (aromatic doublet); pantoprazole 2.584 ppm (methylene); lansoprazole 1.889 ppm (methyl); dexlansoprazole, 1.886 ppm (methyl); tenatoprazole, 3.487 ppm (methoxy)). Intensities were measured as absolute peak heights after phasing and baseline correction using Topspin 3.0. The peak heights were normalized against the peak height measured for the first experiment. All data were collected at 300 K on a Bruker Avance 600 MHz spectrometer equipped with a room temperature probe.

### Chemical shift perturbations

Determination of chemical shift perturbations in purified ^15^N-labeled Tsg101 UEV domain protein complexed to various prazoles was performed as described for tenatoprazole in Strickland, *et al*.^3^ Buffer was exchanged and concentrated using Amicon centrifugal filters (MWCO 3 kDa) into 20 mM potassium phosphate, 50 mM sodium chloride, pH 5.8. Stock solutions of esomeprazole, rabeprazole, pantoprazole, lansoprazole, dexlansoprazole, and tenatoprazole were made using DMSO to a final concentration of 20 mM. 250 µL of ^15^N-Tsg101 (243 µM) was added to 60.75 µL of prazole (20 mM) and 3.189 mL room temperature buffer (20 mM potassium phosphate, 50 mM sodium chloride, pH 5.8) to make a total reaction volume of 3.5 mL, which was left for 16 hours at room temperature. Unreacted prazole and DMSO were removed using Amicon centrifugal filters (MWCO 3 kDa) and buffer containing 20 mM potassium phosphate, 50 mM sodium chloride, pH 5.8. NMR samples (250 µL, Shigemi tubes, 7% D_2_O) were measured at final concentration of the Tsg101-prazole complexes of 200 µM. Chemical shift perturbations were measured as the difference in proton and nitrogen chemical shift between ^1^H/^15^N-heternuclear single quantum coherence experiments before and after binding, using the following equation, as described previously^3^:

$$\Delta \delta =\sqrt{0.5({({H}_{Tsg}-{H}_{Tsg+praz.})}^{2}+{(\alpha ({N}_{Tsg}-{N}_{Tsg+praz.}))}^{2})}$$

where ∆𝛿 is the chemical shift perturbation, *H*_Tsg_, *H*_Tsg+praz_, *N*_Tsg_, and *N*_Tsg+praz._are the proton and nitrogen chemical shifts with and without the addition of the prazole compounds. *α* is calculated as the ratio between the proton and nitrogen chemical shift ranges of the backbone amides of ^15^N-Tsg101 in the free form (*α* = 0.13) as described^42^. Chemical shift perturbation experiments were measured at 300 K on a 600 MHz Bruker Avance spectrometer equipped with a cryoprobe. ^15^N-Tsg101 in the free form and ^15^N-Tsg101 in complex with tenatoprazole resonances were previously assigned using standard triple-resonance experiments. The other Tsg101-prazole complexes were assigned using ^15^N-NOESY experiments measured at 800 MHz and HNCACB experiments measured at 600 MHz (both Bruker Avance equipped with a cryoprobe). Spectra were processed using NMRPipe^43^ and analyzed using CCPN Analysis 2.4.1^44^. The cutoff for large chemical shift perturbations was standardized as 0.084 ppm (1.5 standard deviations from zero for tenatoprazole chemical shift perturbations). Structure figures were rendered using MacPyMOL.

### Prazole effect on virus production

Metabolic activity of the cells in the presence of the prazole drugs for 24 hr was assessed using the WST-1 assay (Roche Applied Science) following the manufacturer's protocol.

The treatment regimens used to assess susceptibility were as follows:

### HIV-1

Cells used: 293 T (ATCC CRL-3216), HeLa (ATCC CCL-2). Methods were as previously described^3^. Briefly, cells were grown in Dulbecco's modified Eagle medium supplemented with fetal bovine serum (10%) and antibiotics (1%) to 70% confluency at 37 °C prior to drug treatment, transfection or toxicity assays. Tissue culture media was aspirated and replaced with control or treatment media prior to transfection unless stated otherwise in figure legends. Transfection was done using XtremeGene reagent (Roche) for DNA. Plasmids pNL4-3ΔEnv, pIIIB-Env3-1, pCMV-Gag-HA encoding HIV-1 Gag C-terminally tagged with hemagglutinin were previously described^3^. For production of virus particles, cells were transfected with pNL4-3-ΔEnv and pIIIB Env3-1 plasmids and for production of virus-like particles (VLP), cells were transfected with Gag-encoding construct as previously described^3^. After 24 hr, tissue culture media was collected and passed through a 0.45 micron filter; cells were scraped with a rubber policeman, rinsed with PBS and pelleted. For virus or VLP isolation, filtered media was centrifuged through a 20% sucrose cushion at 22,000 × g for 90 min at 5 °C and the pellet fraction saved for analysis. For cell lysate preparation, cell pellets were lysed with Triton X-100 buffer (50 mMTris, pH 7.4, 137 mM NaCl, 1.5 mM MgCl_2_, 1 mM EDTA, 1% Triton X-100) containing a protease inhibitor cocktail. VLP and cell lysate samples were analyzed by Western blotting. Primary antibodies used were: Rabbit anti-CA; anti-actin (Sigma); anti HA (Biolegends). Secondary antibodies used were: goat anti-mouse IgG Alexa Fluor 680 (Molecular Probes); goat anti-rabbit IRDye800 (Rockland). Protein bands were visualized using an infrared-based imaging system (Odyssey, LI-COR Biotechnology) and band intensities measured using the Li-Cor Odyssey software version 2.1.15.

### EBV

CLIX-FZ cells were derived from the endemic EBV-positive Burkitt lymphoma cell line HH514-16 as described previously and used in lytic virus production following (re)activation from latency via induction of exogenous ZEBRA expression by addition of doxycycline (5 μg/ml). CLIX-FZ cells were treated with DMSO, doxycycline plus DMSO or doxycycline plus 20 µM or 40 µM tenatoprazole. After a 72 hr incubation period, cells and conditioned media were harvested. EBV particles in the media were pelleted, washed with 1X PBS, treated with DNase and processed for measurement of extracellular EBV DNA. Cells were lysed and total DNA extracted and used to measure intracellular EBV DNA. Relative viral DNA copy number from each preparation was determined using quantitative-PCR (qPCR) to amplify the EBV *BALF5* gene with forward primer CGTCTCATTCCCAAGTGTTTC and reverse primer GCCCTTTCCATCCTCGTC. *Nuclear and cytosolic fractionation*: Cells were washed with 500 µl of ice-cold 1 × PBS, pellets were resuspended in 500 µl of ice-cold 1X nucleus isolation Buffer (#7006, Cell Signaling Technology), and incubated at 4 °C for 1 hr. Nuclei were pelleted by centrifugation at 2000 × g at 4 °C for 5 min and resuspended in 500 µl ice-cold 1X nucleus lysis buffer (#7007, Cell Signaling Technology) after carefully separating the supernatant containing the cytosolic fraction. Nuclear and cytosolic fractions were treated with 10 mg/ml of proteinase K (catalog no. 19131; Qiagen) at 56 °C overnight. The enzyme was inactivated by heating at 95 °C for 1 hr. Aliquots of fractions were used to amplify the EBV *BALF5* gene, mitochondrially encoded *Cytochrome B* (*CYTB*) gene, and nuclear *KAP1* gene by qPCR. Relative numbers of EBV genomes were calculated using the delta-delta CT method by normalizing to *KAP1* and *CYTB* genes in nuclear and cytosolic fractions, respectively.

### EBOV

Hela cells were pre-treated with media containing either DMSO or ten doses of tenatoprazole up to 100 μM, repeated four times on a single plate. After 2 hr, cells were infected with EBOV (Zaire), at MOI = 0.5. Infection was stopped after 24 hr by fixing cells with a formalin solution. Infected cells were detected by immuno-staining viral glycoprotein (GP) with anti-GP antibodies and the signal for GP-staining converted into % infection. Viability was determined from the number of nuclei per well in comparison to infected but untreated controls. Two independent experiments were conducted.

### DENV

Confluent monolayers of Vero cells or 293 T in a 12-well format were treated with tenatoprazole at concentrations of 0, 10 μM, 50 μM and 250 μM in 1 μL of DMSO added to 500 mL of 5% BCS DMEM. Cells were incubated for 6 hr at room temperature while being rocked gently. Following treatment, the medium was aspirated and replaced with 250 µL containing 100 FFU (MOI = 0.0001) or 10,000 FFU DENV2^syn^ (MOI = 0.01) as described^45^. The plates were rocked at room temperature for 30 minutes, then 250 µL of 5% BCS DMEM was added to a total volume of 500 µL. The plates were then incubated at 37 °C, 5% CO_2_ for 2 hr. The medium in each well was aspirated and replaced with 1 mL of 0.6% Tragacanth Gum in 5% BCS DMEM and incubated at 37 °C, 5% CO_2_ for 5 (D2-Syn) days. The D2-Syn focus forming assay was developed with 4g2 (primary anti-flavivirus antibody hybridoma supernatant) that recognizes a conserved epitope in Flavivirus envelope protein, anti-mouse HRP conjugated secondary antibody (GeneTex), and HRP substrate (Vector VIP). Synthetic Dengue virus type 2 strain 16681 (D2-Syn) acquired from the laboratory of Dr. Eckard Wimmer at Stony Brook University.

### ZIKV

Confluent 293 T cells were pre-treated for six hours with media containing DMSO (0), 10 µM, 50 µM, or 100 µM tenatoprazole. Media was aspirated and the cells infected with ZIKV PRVABC59 at a concentration of 2 × 10^4^ FFU/mL in a volume of 500 µL (MOI ~0.01). After two hours of infection, media was replaced with fresh treatment media. After five days, conditioned media was collected and measured for virus titer by plaque assay. Vero cells in 12-well plates were infected with virus in serially diluted samples for two hours, layered with 0.6% Tragacanth Gum in 5% BCS DMEM and incubated at 37 °C, 5% CO_2_ for five days. Plaques were visualized by staining with crystal violet. ZIKV strain PRVABC59 was provided by BEI Resources (NIAID, NIH: Zika Virus, PRVABC59, NR-50240). PRVABC59 is a recent isolate (2015) from a human patient in Puerto Rico^46^.

### MAYV

293 T cells at ~80% confluency in 6-well plates were pre-treated for 6 hours with media containing either DMSO (0), 6.25 µM, 12.5 µM, 25.0 µM, 50 µM, or 100 µM tenatoprazole. Media was then aspirated, and the cells were infected with MAYV TRVL 15537 (ATCC VR-1277) at a concentration of 2 × 10^3^ FFU/mL in a volume of 500 µL (MOI ~0.001). After two hours of infection, media was replaced with fresh treatment media. After two days, conditioned media was collected and measured for virus titer by plaque assay. Vero cells in 12-well plates were infected with virus in serially diluted samples for two hours, layered with 0.6% Tragacanth Gum in 5% BCS DMEM and incubated at 37 °C, 5% CO_2_ for five days. Plaques were visualized by staining with crystal violet.

### PV

293 T cells were pre-treated for 7 hr with media containing DMSO (0), 20 µM, or 100 µM tenatoprazole. The cells were then infected with 10,000 PFU of the Mahoney strain of poliovirus (MOI = 0.01). After two hours of infection, media was aspirated and replaced with fresh treatment media. After 24 hr, conditioned media was collected and measured for virus titer by plaque assay. Vero cells in 12-well plates were infected with virus in serially diluted samples for two hours, layered with 0.6% Tragacanth Gum in 5% BCS DMEM and incubated at 37 °C, 5% CO_2_ for five days. Plaques were visualized by staining with crystal violet. Poliovirus Type I Mahoney was acquired from the laboratory of Dr. Eckard Wimmer at Stony Brook University.

## Supplementary information

Supplemental information.
