# Supplementary material for: Selective Targeting of Virus Replication by Proton Pump Inhibitors
Source: Sci Rep. 2020 Mar 4;10:4003. doi: 10.1038/s41598-020-60544-y (PMC7055211; doi:10.1038/s41598-020-60544-y)
Supplement: Supplementary file 1 — Supplemental information. [file 41598_2020_60544_MOESM1_ESM.pdf]

# Selective Targeting of Virus Replication by Proton Pump Inhibitors

Susan M. Watanabe[a], Lorna S. Ehrlich[a], Madeleine Strickland[b], Xiaofan Li[c], Veronica Soloveva[d], Arthur J. Goff[d], Charles B. Stauft[a], Sumita Bhaduri-McIntosh[c], Nico Tjandra[b] and Carol Carter[a#]

[a] Department of Microbiology and Immunology, Stony Brook University, Stony Brook, NY 11794-5222, USA; [b]Laboratory of Molecular Biophysics, National Heart, Lung, and Blood Institute, National Institutes of Health, Bethesda, MD, 20892, USA; [c] Department of Pediatrics, Division of Infectious Diseases and Department of Molecular Genetics and Microbiology, University of Florida, Gainesville, FL 32610, USA; [d]U.S. Army Medical Research Institute of Infectious Diseases, Frederick, MD, 21702-5011, USA.

[#]Corresponding authors:

Carol Carter: Department of Microbiology and Immunology, School of Medicine, Stony Brook University, Stony Brook, NY 11794-5222, USA; Tel. (631) 632-8801; email: carol.carter@stonybrook.edu

Nico Tjandra: Laboratory of Molecular Biophysics, National Heart, Lung, and Blood Institute, National Institutes of Health, Bethesda, MD, 20892, USA; Tel. (301) 402-3029; email: tjandran@nhlbi.nih.gov

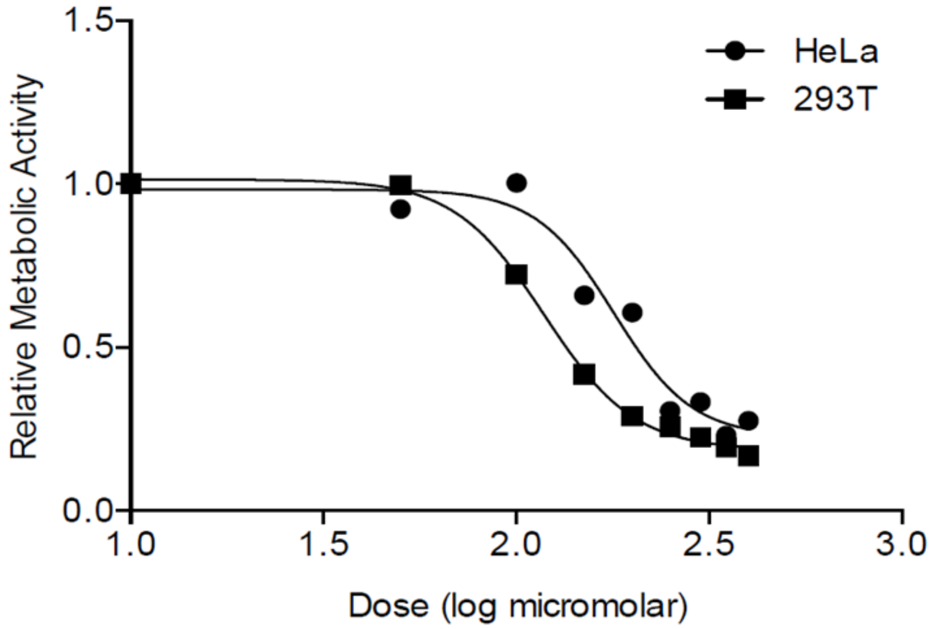


**Supplementary Figure S1.** Tenatoprazole effect on HeLa and HEK-293T cellular metabolism. Toxicity was determined by measuring metabolic activity (Roche, Cell Proliferation Reagent WST-1) after cells were grown for 24 hr with the drug. The $CC_{50}$ values determined for Hela and 293T cells were 129 $\mu$M and 123 $\mu$M, respectively (Prism 6, Graph Pad Software Inc) with a goodness of fit R square values of 0.97 and 0.99, respectively.

| Compound | Observed/Da | Calculated/Da |
|---|---|---|
| ● Rabeprazole | 359.1304 | 359.1303 |
| ● Monomer 1 | 343.1335 | 343.1354 |
| ● Monomer 2 | 368.1307 | 368.1848 |
| ● Monomer 3 | 339.1041 | 339.1583 |
| ● Dimer 1 | 684.2552 | 684.2552 |
| ● Dimer 2 | 716.2277 | 716.2451 |

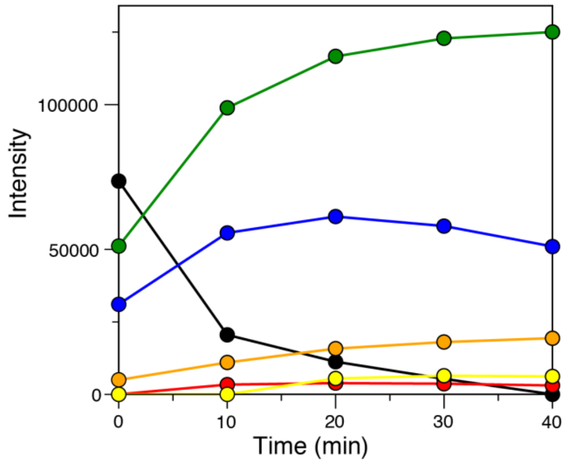


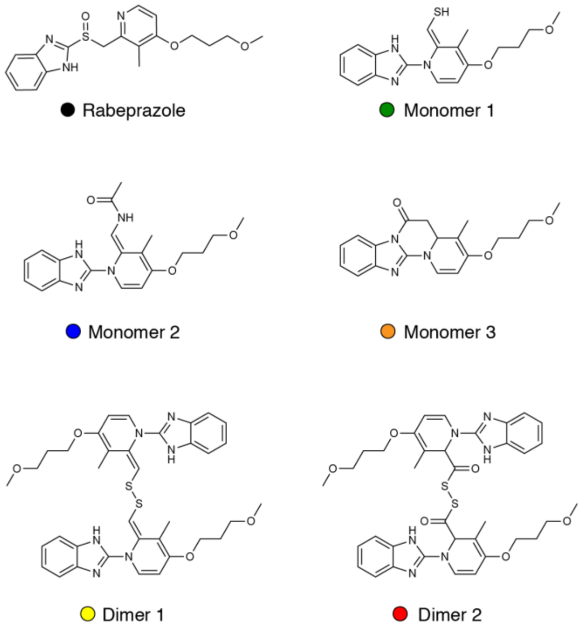


**Supplementary Figure S2.** Prodrug conversion to activated prazole. The formation of activated prazole was monitored using Ellman's reagent, which turns orange when it comes into contact with thiol (S-H) groups. Mass spec was used to detect the product in a time-dependent manner. Loss of prodrug correlates with the appearance of an active form. The molecules shown are suggestions likely to have the same molecular formula as the actual compounds.

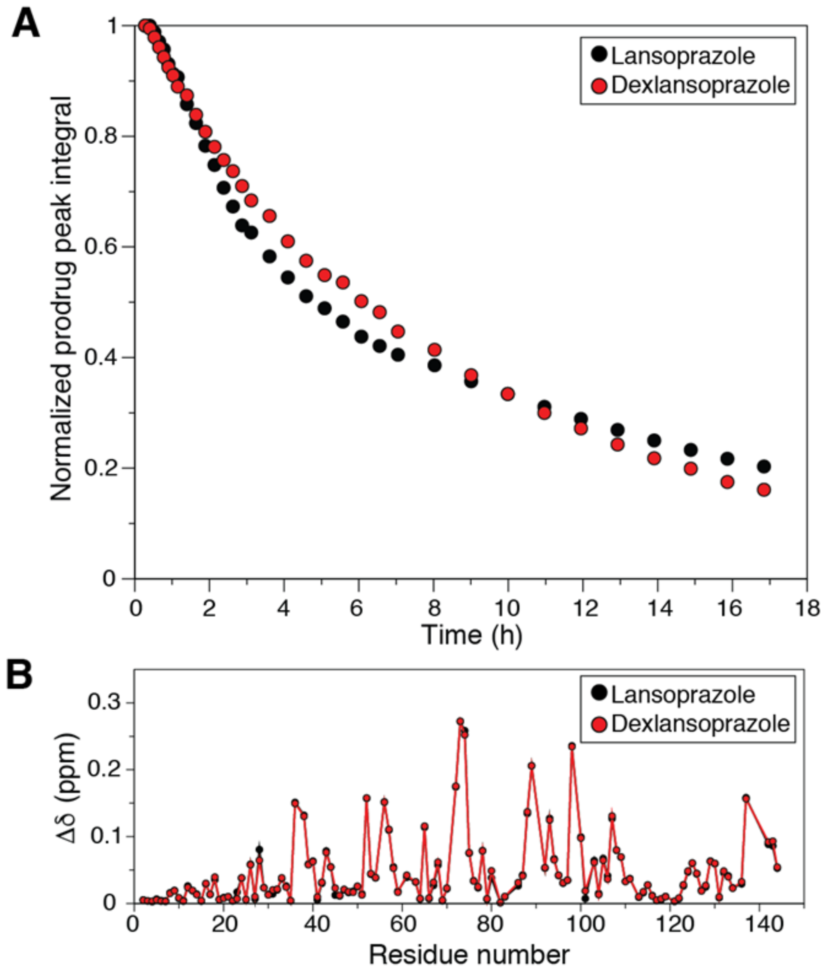


**Supplementary Figure S3**. Prazole degradation rate and chemical shift perturbations in Tsg101 UEV for racemic lansoprazole and single enantiomer dexlansoprazole. *Panel A*, Prodrug remaining at time intervals over a 17 hr examination period, measured using 1D NMR spectra. Peak heights were normalized to the initial reading for each prodrug and plotted against time. *Panel B*, Chemical shift perturbations in Tsg101 UEV complexed with lansoprazole and dexlansoprazole.

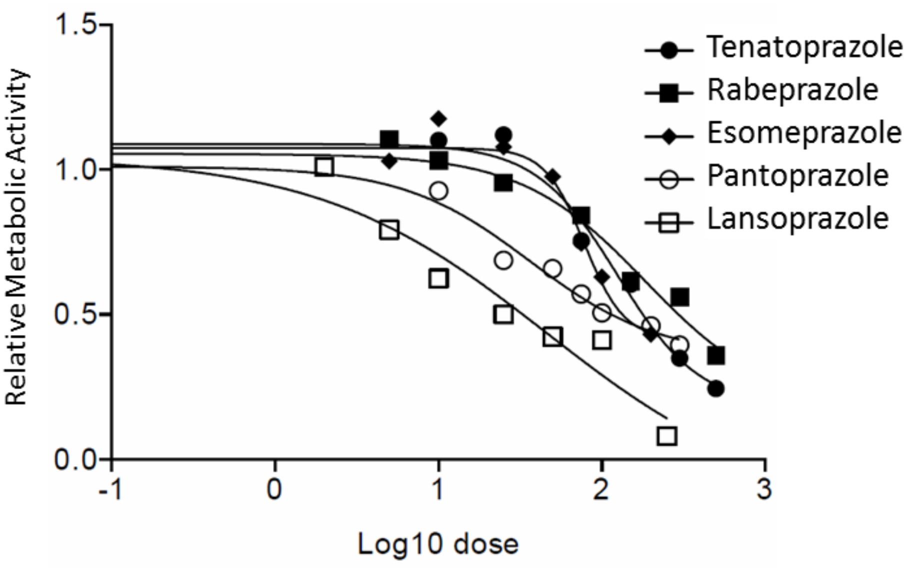


**Supplementary Figure S4:** Cellular metabolism measurements for tenatoprazole, esomeprazole, pantoprazole, rabeprazole, and lansoprazole. Metabolic activity was measured with a colorimetric assay (Roche, Cell Proliferation Reagent WST-1) after cells were grown for 24 hr with a drug. The $CC_{50}$ values were: 125 μM (tenatoprazole), 150 μM (rabeprazole), 75 μM (esomeprazole), 40 μM (pantoprazole), 50 μM lansoprazole) as calculated by Prism 6, Graph Pad Software, Inc. The goodness of fit R square values varied from 0.94 to 0.97.

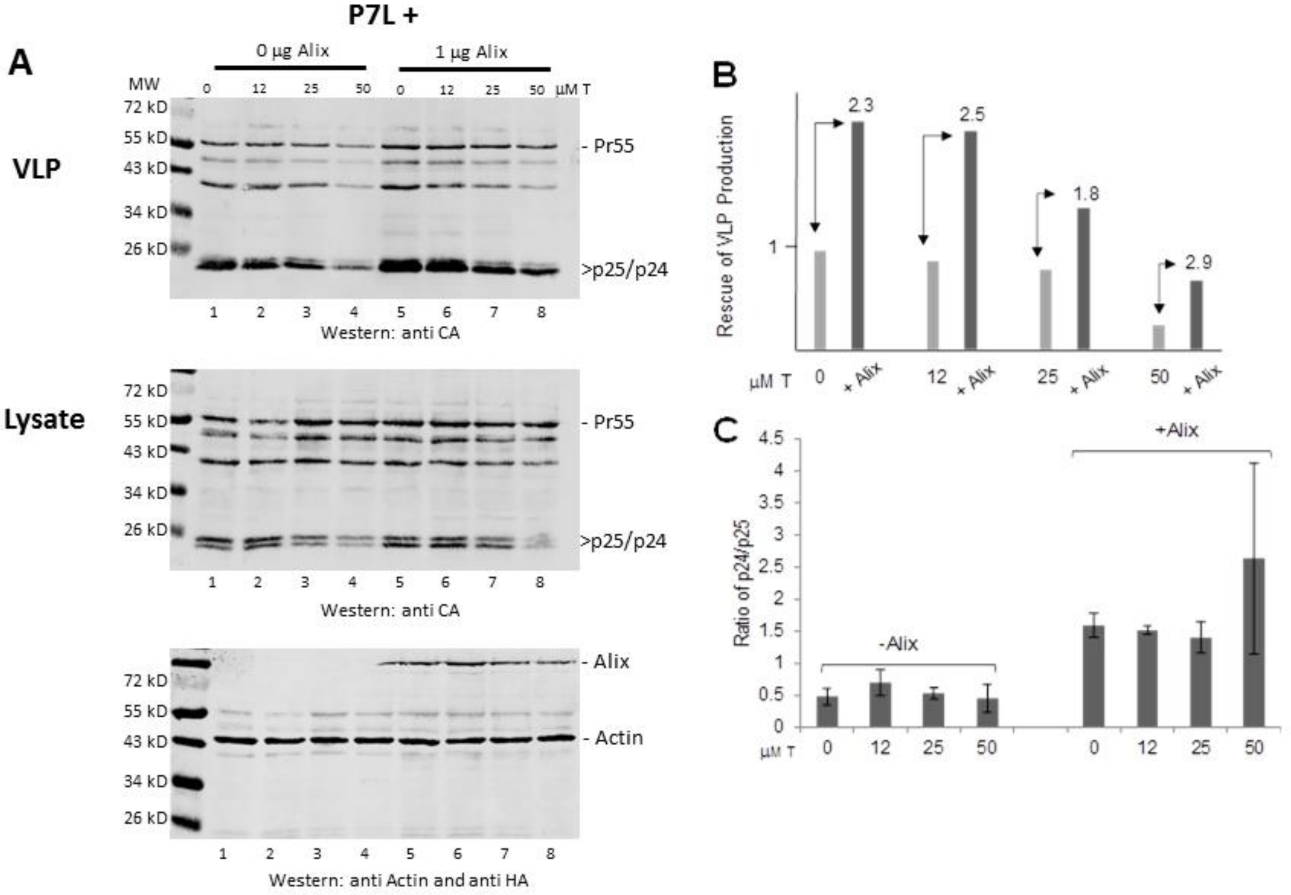


**SUPPLEMENTARY FIGURE S5**. Tenatoprazole effect on Alix-mediated rescue of pNL4-3-P7L-Gag release. ***Panel A***, 293T cells were treated with DMSO (lane 1) or tenatoprazole (Lanes 2 to 4) six hours prior to transfection with DNA encoding pNL4-3-P7L-Gag (lanes 1-8) and DNA encoding HA-tagged Alix (lanes 5-8). Western blot analysis of viral particles (*top*) and cell lysates (*bottom*) for CA p24-related proteins, actin and Alix. ***Panel B****,* Quantitative analysis of Alix rescue of VLP production. VLP release efficiency was determined in the absence and presence of Alix and normalized to cellular Gag accumulation plus VLP-associated Gag (*i.e.*, VLP/(VLP + cellular Gag). ***Panel C,*** Quantitative analysis of capsid maturation efficiency based on CAp25/p24 signals in the cell lysate.
